# Parliament and the Profession

**Published:** 1915-12-04

**Authors:** 


### PARLIAMENT AND THE PROFESSION.

### First Eastern General Hospital.

In the House of Commons on November 25 Mr. Tennant stated he had received a representation concerning the management of the 1st Eastern General Hospital, Cambridge, and that he had caused it to be referred for inquiry and report.

### The Scottish Midwives Bill.

In moving the second reading of the Midwives (Scotland) Bill on November 25 Mr. McKinnon Wood said that as a result of the depletion of the medical profession rural districts had been left inadequately provided with practitioners so that competent midwives were absolutely necessary throughout Scotland. The Bill was committed to a Committee of the whole House.

### Scottish Pharmacists and the Drug Tariff

The existing drug tariff is to continue in force in Scotland until July 1, 1916, owing to representations made on behalf of Scottish Panel Committees that the proposed new tariff is inequitable. In England and Wales, however, the new tariff will come into force at the beginning of the year. This was the gist of a lengthy statement made by Mr. Charles Roberts in the House of Commons.

### Wasting the Time of the House.

Why was a parcel fully addressed to a private in a French hospital returned to the sender marked "Present location uncertain," seeing that the said private was then a patient in the hospital? Obviously there was a mistake somewhere, but surely this is not a matter of sufficient importance for discussion in Parliament. Yet on November 24 the Postmaster-General had to account for this trivial accident. Let us get on with the war!

### A Case for More Inquiry.

A question asked in the House on November 22 suggested that men who have been passed as medically fit on enlistment had, in many instances, been discharged after partial recovery from wounds received in action, on the ground that they were medically unfit for further service by reason of the alleged existence of disease prior to enlistment, and that in consequence the men were deprived of their claim to pensions which otherwise would have been granted to them. Mr. Tennant stated, however, that no such case had come to his notice.

### Tuberculous Soldiers.

In a question asked on November 25 Mr. R. McNeill alleged that many soldiers are invalided home from the Front suffering from tuberculosis for whose cure no proper provision is made. He desired to know whether the Insurance Commissioners could place at the disposal of the War Office, for the exclusive reception of tuberculous soldiers, one or more of the sanatoria at present empty. Mr. Charles Roberts, in his reply, explained that special arrangements are in operation which secure that the necessary accommodation is made available without delay.

### Officers of the R.A.M.C. in Egypt.

In the House of Commons on November 23 Mr. Watt asked the Financial Secretary to the War Office whether the Colonial allowance of 3s. a day was paid in Egypt to all officers of the R.A.M.C. whose stay in Egypt exceeded fourteen days; whether this applied to temporary lieutenants there whose stay in that country often extended to six months or a year; if not, would he say why this exception was made to this particular class of officer. Mr. Forster replied that temporary lieutenants served under a special contract at a rate of pay explicitly fixed to cover all cash allowances.

### The Irish Apothecaries Again.

In the course of a question addressed to the Under-Secretary for War on November 24, Mr. Byrne mentioned that Dr. C. B. Maunsell had resigned his position as examiner in surgery to the Apothecaries' Hall of Ireland as a protest against the attitude of the General Medical Council with reference to the Hall (The Hospital, November 27, p. 190). He also alleged that no other Dublin surgeon could be induced to accept the office under "the present conditions of espionage," with the result that it was impossible for this medical licensing board to qualify men for the R.A.M.C.

### Unvaccinated Recruits.

Asked to quote the wording of any regulation stipulating that those who join the Army must consent to vaccination, Mr. Tennant referred his questioner to paragraph 117 of the Recruiting Regulations—namely: "The enlistment of a man who states on attestation that he is unwilling to be vaccinated will not be proceeded with," and to paragraph 515 of the Regulations for Army Medical Services, which runs as follows: "With the exception of those bearing distinct marks of smallpox, all recruits, including the Special Reserve, will be vaccinated on the second day after joining the depôt."

### Who Advises Mr. McKenna?

On November 24 Mr. Peto called attention to the scarcity and high price of aspirin, salicylic acid, phenacetin, antipyrin, and other drugs of the coal-tar series. Mr. McKenna said that the position as regards the available supplies of the needed drugs was on the whole improving, and that there was no reason to anticipate any serious shortage in the future. Where does the Chancellor of the Exchequer get his information about the supply of drugs? The price of phenacetin is now twenty times the pre-war figure, and is advancing almost every day; antipyrin is ten times the price quoted before the war, and some drugs are almost unobtainable; yet Mr. McKenna tells the House that "the position as regards available supplies is on the whole improving"!

### Army Medical Services Advisory Board.

Replying to a question as to the constitution of the Advisory Board of the Army Medical Services, Mr. Tennant stated that there are eleven members—seven military and four civilian. The civilian members are paid £200 a year each. Two military members receive extra pay of £150 a year; five receive nothing for this duty. No formal meetings have been held in 1915, but all the paid members have been constantly consulted and have given very valuable advice throughout the war. A further series of questions on the same subject was asked on November 25, and in reply to Mr. Shirley Benn, Mr. Tennant said that he was not aware that there had been any want of unanimity amongst the members save that which is usual and a healthy symptom in mobile minds.

